# PSYCHOMETRICS IN THE DEVELOPING OF LONGITUDINAL MEASURES OF FRAILTY

**DOI:** 10.1093/geroni/igac059.2999

**Published:** 2022-12-20

**Authors:** Stacey Voll, Graciela Muniz Terrera, Scott Hofer

**Affiliations:** University of Victoria, Victoria, British Columbia, Canada; Ohio University, Athens, Ohio, United States; University of Victoria, Victoria, British Columbia, Canada

## Abstract

Frailty has become an increasingly important aspect in the field of health research in studies of vulnerability to adverse events and individuals functioning due to accumulated health deficits. Yet, there is no agreement on how to measure frailty or identify adults as frail, resulting in high heterogeneity between estimates of frailty and identification of frail individuals. The purpose of this poster is to provide a summary of the process in the development of a frailty index with robust psychometric properties to be used in research using longitudinal health study sets.

